# Prediction of Bronchopulmonary Dysplasia in Preterm Infants Using Postnatal Risk Factors

**DOI:** 10.3389/fped.2020.00349

**Published:** 2020-06-26

**Authors:** Li Ding, Huawei Wang, Haifeng Geng, Ningxun Cui, Fengxia Huang, Xueping Zhu, Xiaoli Zhu

**Affiliations:** ^1^Department of Neonatology, Children's Hospital of Soochow University, Suzhou, China; ^2^Department of Intervention, The First Affiliated Hospital of Soochow University, Suzhou, China

**Keywords:** bronchopulmonary dysplasia, preterm infant, sB7-H3, IL-18, neonatal critical illness score

## Abstract

**Objective:** To identify postnatal risk factors for bronchopulmonary dysplasia (BPD) development in preterm infants with gestational age ≤32 weeks.

**Methods:** Seventy-two preterm infants(30 with BPD and 42 non-BPD controls) admitted in the neonatal intensive care unit (NICU) of the Children's Hospital of Soochow University during 2017 were enrolled in this prospective longitudinal study. Perinatal clinical data, a neonatal critical illness score (NCIS), different soluble B7-H3(sB7-H3), and interleukin-18 (IL-18) levels by days after birth were collected. An early predictive model for BPD development was established based on clinical data using multiple logistic regression analysis. And the sensitivity and specificity of the model were assesed by ROC curve.

**Results:** Electrolyte disturbances, hemodynamically significant patent ductus arteriosus (hs-PDA), and the age that infants achieved 120 kcal/kg.d via enteral feeding ≥40 days after birth were found to be associated with the BPD pathogenesis. Serum sB7-H3, IL-18, and NCIS were significantly higher in the BPD group compared to the non-BPD group (*p* < 0.05). BPD group had significantly lower enteral fluid and caloric intake compared to the non-BPD group at 1, 7, 14, and 28 days after birth. The risk factors were analyzed by multiple logistic regression and a predictive model of a combination of sB7-H3 (day 7), IL-18 (day 14), NCIS, and clinical risk factors was evaluated via ROC curve with an area under the curve (AUC) of 0.960 having sensitivity of 86.7% and a specificity of 97.6%, respectively.

**Conclusion:** The causes of BPD are multifactorial postnatal risk factors. And the combination of sB7-H3 (day 7), IL-18 (day 14), NCIS, and clinical risk factors (electrolyte disturbances, hs-PDA, and the age that infants achieved 120 kcal/kg.d via enteral feeding ≥40 days after birth) might be served as an optimal predictive model for the occurrence of BPD.

## Introduction

Bronchopulmonary dysplasia (BPD) is a chronic lung disease mainly affecting preterm infants who require respiratory support at birth. Despite the advanced perinatal and neonatal care, the morbidity, and mortality of BPD remains stable (1, 2). The detrimental effects of BPD on the respiratory and nervous system could last into adulthood, seriously impacting the well-being of surviving children (3, 4). Approximately 45% of preterm infants born at a gestational age of 29 weeks develop BPD. Although the survival rate of extremely preterm infants has increased, the incidence of BPD has also increased over the past few decades (5).

The mechanisms of BPD are still unclear and specific treatment is lacking. Prevention and early diagnosis are essential. Pathogenesis of BPD involves multiple factors (6, 7), including immature lung tissue, excessive inflammatory damage, and an abnormal repair process after injury (8–10). sB7-H3 is a novel member of the B7 superfamily and is important in the regulation of TLR2-mediated immune responses and innate immunity (11). Toll-like receptor 2 (TLR2) signaling is enhanced in hyperoxic fetal lung fibroblasts *in vitro*. The induction of TLR2 signaling in fetal lung fibroblasts may contribute to the pro-inflammatory state in the preterm neonate on supplemental oxygen (12). Therefore, sB7-H3 may be a possible factor that promotes the occurrence of BPD. IL-18, a proinflammatory mediator and member of the IL-1 cytokine family, is critical in pulmonary diseases, such as acute respiratory distress syndrome (ARDS) (13) and chronic obstructive lung disease (COPD) (14). IL-18 can participate in the progression of pulmonary inflammation by promoting the type I helper T cells (Th1) response and can promote the proliferation of fibroblasts and collagen precipitation in the process of pulmonary fibrosis (15). Therefore, IL-18 may be critical for the occurrence and development of BPD and acts as a possible biomarker for BPD prediction.

Perinatal clinical data, a neonatal critical illness score (NCIS), different soluble B7-H3(sB7-H3), and interleukin-18 (IL-18) levels by days after birth were collected to identify postnatal risk factors that predict the occurrence of BPD in preterm infants within a gestational age ≤32 weeks.

## Methods

### Study Design

Infants admitted to the neonatal intensive care unit (NICU) of the Children's Hospital of Soochow University from January 01, 2017, to December 31, 2017 were enrolled in this study. The inclusion criteria were infants with gestational age ≤32 weeks and hospital stays ≥28 days. The exclusion criteria included the age more than 24 h upon admission to the NICU, infection at admission, congenital abnormalities or inborn errors, surgery intervention requirement during the hospital stay, and/or incomplete clinical data.

This study was approved by the ethics committee of Children's Hospital of Soochow University. Informed consent was obtained from all the parents of the included infants Under their permission.

### Feeding Principles

Enteral feeding, minimal feeding, and even non-nutritive sucking should begin as soon as possible within postnatal 24 h. Positive and individualized principles should be addressed either in enteral or parenteral nutrition administration.

1. As donated breast milk was unavailable in our unit, mothers were encouraged to feed their babies with expressed breast milk (EBM). If the EBM was unavailable, infants were fed with formula.
2. If feeding intolerance occurs, minimal feeding, and/or non-nutritive sucking would be conducted instead of suspending feeding. Once the related symptoms were improved, the feeding would be continued. The choices of formula types, such as hydrolyzed protein formula, low lactose formula, or lactose addition formula, were based upon patients' clinical conditions. The total caloric requirement would be supplemented through parenteral nutrition.
3. The speed of feeding advancement was 20–25 ml/kg.d.
4. When the amount of breastfeeding reached 50–80 ml/(kg.d), breast milk fortifier would fortify by 1/2. If the infant was intolerant, the proportion or even fortify by 1/3–1/4, and then gradually increased the dose according to the clinical situation.

### Collection of Clinical Data

The following data were collected from hospital records: (1) general condition of the infant, including infant gender, gestational age (according to the first day of the last menstrual period) at birth, birth weight, mode of delivery, age at admission, *in vitro* fertilization, multiple birth, small for gestational age, Apgar Score ≤7 (1 or 5min), prealbumin ≤80 mg/L, and albumin ≤30 g/L; (2) maternal conditions, including age, pregnancy induced hypertension, gestational diabetes, abortion ≤2 times, oligohydramnios, placental abruption, placenta previa, and antenatal corticosteroids use; (3) birth injuries, conditions, and comorbidities present in the infant, including NRDS, ventilator associated pneumonia (VAP), pneumothorax, CNS, periventricular/intraventricular hemorrhage (PVH/IVH), PVL, parenteral nutrition associated cholestasis (PNAC), liver damage, hemodynamically significant PDA(hs-PDA), neutropenia, anemia, retinopathy of prematurity (ROP), neonatal hypoglycemia, and electrolyte disturbances; (4) treatment modalities applied during hospitalization of the preterm infant, including invasive ventilation, duration of invasive ventilation, ventilator mode (normal frequency or high frequency), days of oxygen inhalation, time of blood transfusion, the age when enteral feeding was initiated, the age when goal energy intake (120 kcal/kg.d) was reached by enteral feeding, and rate of weight gain; (5) feeding of the preterm infant, including oral fluid intake, intravenous fluid intake, enteral caloric intake, intravenous caloric intake, and weight gain (percentage of birth weight) on 3, 7, 14, and 28 days after birth; and (6) the neonatal critical illness score (NCIS) on the admission day of the preterm infant.

We firstly define the important clinical indicators for the sake of understanding.

1. Diagnosis of BPD and Clinical Grading (16)
  The diagnostic criteria of BPD adopted in our study was based on the standard of the National Institute of Child Health and Human Development (NICHD) published in 2001, which defines BPD as follows: (i) preterm low birthweight infants treated with oxygen (FiO_2_ >0.21) for at least 28 days; (ii) persistent or progressive respiratory insufficiency; (iii) lungs with typical X-ray or CT scan findings (e.g., bilateral lungs with enhanced texture, reduced permeability, ground glass-like, localized emphysema, or cystic changes); (iv) exclusion of congenital cardiopathy, pneumothorax, pleural effusion, and sputum. The clinical grading was based on the supplemental O_2_ of the infants at 36 weeks postmenstrual age or discharge (GA <32 weeks) and at 56 days postnatal age or discharge (GA ≥32 weeks). The clinical grading was classified as follows: mild: breathing room air; moderate: a fraction of inspired oxygen (FiO_2_) <0.3; severe: FiO_2_ ≥0.3 and/or positive pressure ventilation or mechanical ventilation.
2. Diagnosis of VAP (17)
  VAP was defined as a nosocomial infection hanppening 48 h after mechanical ventilation.
3. Diagnosis of NRDS (18)
  NRDS was defined as the presensce of respiratory distress and increased oxygen requirement (FiO_2_ >0.4), which can not be explained by other causes via chest x ray and lab findings.
4. Diagnosis of PVH/IVH (19)
  Sonographic findings of PVH/IVH were graded into three categories based on McMenamin's classification: Grade I: subependymal hemorrhage with minimal or no IVH; Grade II: IVH, but neither lateral ventricle completely filled with blood, with or without mild ventricular dilatation; and Grade III: IVH completely filling and distending at least one lateral ventricle.
5. Diagnosis of PNAC (20)
  PNAC was defined as cholestasis attributable to PN use, with other parameters excluded.
6. Diagnosis of Electrolyte disturbance
  Electrolyte disturbance included hyponatremia (serum sodium <135 mmol/L), hypernatremia (serum sodium >150 mmol/L), hypokalemia (serum potassium <3.5 mmol/L), hyperkalemia (serum potassium >5.5 mmol/L), hypocalcemia (serum calcium <2.2 mmol/L), hypercalcemia (serum calcium >2.7 mmol/L), hypochloremia (serum chloride <95 mmol/L), and hyperchloremia (serum chloride >105 mmol/L).
7. Diagnosis of NCIS (21)
  NCIS was defined by Chinese Medical Association Emergency Branch Pediatrics Group, Chinese Medical Association Pediatric Branch Emergency Department, Neonatal Group. It includes heart rate (HR), systolic blood pressure (SBP), arterial partial pressures of O_2_ (PaO_2_), pH, respiration, blood sodium, blood potassium, blood urea nitrogen, creatinine (Cr) levels, hematocrit, and gastrointestinal bleeding/bloating. NCIS Scores >90 are non-critical, 70–90 are critical, and <70 are extremely critical. NCIS is important for determining prognosis and guiding newborns' treatment and prognosis.
8. Diagnosis of hs-PDA (22)
  Echocardiographic evidence of a hs-PDA met one of the following criteria: ductal diameter≥1.5 mm, unrestrictive pulsatile ductal flow (ductus arteriosus peak velocity <2.0 m/s), left heart volume loading (eg., left atrium to aortic ratio>1.5), left heart pressure loading (eg, early passive to a late atrial contractile phase of transmitral filling ratio >1.0 or isovolumic relaxation time ≥50).

#### ELISA of sB7-H3 and IL-18

The puncture of the peripheral vein was performed on days 1, 7, 14, and 28 after admission and 1–1.5 ml of peripheral venous blood was collected. The blood was centrifuged at 4°C and 3,000 rpm for 5 min. The supernatant was removed and stored in 2–3 separate Eppendorf tubes in −80°C until use. All blood samples were either processed immediately after collection or stored in 4°C and processed within 6 h. The levels of sB7-H3 and IL-18 in the serum was quantified with corresponding ELISA kits (R&D Systems, USA) following the manufacturer's instructions.

### Statistics

Categorical variables were expressed as percentage and processed with either χ^2^-test or Fisher's exact test while normal distributed variables were expressed as mean ± sdandard diviation(m ± sd) and processed by independent *t*-test or one-way analysis of variance (ANOVA), variables did not comply with normal distribution were expressed as median (eg., 25th percentile, and 75th percentile) and non-parametric test such as MU and KW were adopted for statistical analysis. Significant factors were picked out by *p* < 0.05 and recruited in logistic regression to determine the risk factors of patients having BPD. A receiver operating characteristic curve (ROC) was drawn to evaluate the predictive power of the risk factors by obtaining the area under the curve (AUC). Youden's J statistic (Youden's index = sensitivity + specificity – 1) was used to calculate the cut-off points. A *p* < 0.05 was considered As statistical significant. All statistical analyses were carried out using SPSS 17.0 software.

## Results

### The General Characteristics of Preterm Infants

From January 2017 to December 2017, 91 infants were involved in by total and 3 were excluded due to short hospital stay while 2 were out owing to surgery during hospitalization. Among the remaining 86 infants, 30 were diagnosed with BPD. Another 56 infants were non-BPD patients, among which, 14 were excluded attributed to incomplete clinical data collection and non-standard blood samples. Eventually, 42 infants were classified as non-BPD group (Appendix 1). The gender distribution of enrolled patients were male predominant (44 cases, 61.11%). The birh weight ranged from 740 to 2,050 g(1355.56 ± 271.30 g). The gestational age ranged from 26 + 3 to 32 weeks (30.06 ± 1.39 w), In the BPD group, 3 had mild BPD, 22 had moderate BPD and 5 had severe BPD.

### Risk Factors for BPD in Preterm Infants

PVH/IVH (III-IV degree), PNAC, hs-PDA, electrolyte disturbance, invasive ventilation, days with FiO_2_ >0.4, and blood transfusion≥ 3 times were significantly more common in the BPD group (*p* < 0.05) (The cut-offs of “days of FiO_2_ >0.4” and “blood transfusion ≥3 times” were decided based on the data). Compared with non-BPD group, the BPD group was more later on enteral feeding and with a higher rate of the age that infants achieved 120 kcal/kg.d via enteral feeding ≥40 days after birth (*p* < 0.05; Table 1) (the cut-off was decided based on the data).

**Table 1:** Clinical characteristics of the preterm infants^*^ and their mothers.

	**BPD group (*n* = 30)**	**Non-BPD group (*n* = 42)**	**χ^2^/U/t**	***P***
Male (%)	17 (56.67)	27 (64.29)	0.427	0.513
Gestational age (weeks)	29.76 ± 1.54	30.28 ± 1.24	1.581	0.118
Weight (g)	1305.00 ± 315.76	1391.67 ± 231.81	1.344	0.183
Cesarean (%)	11 (36.67)	14 (33.33)	0.086	0.770
Time of admission (h)	2.00 (1.50,4.50)	2.25 (1.00,4.00)	609.000	0.808
*In vitro* fertilization (%)	4 (13.33)	5 (11.90)	0.033	0.857
1 gravida 1 para (%)	8 (26.67)	14 (33.33)	0.367	0.545
Twins and multiple births (%)	9 (30.00)	10 (23.81)	0.345	0.557
Less than gestational age (%)	4 (13.33)	3 (7.14)	——	0.440^F^
Apgar Score ≤7(1 or 5min) (n,%)	14 (46.67)	11 (26.19)	3.237	0.072
Prealbumin ≤80 mg/L (%)	5 (16.67)	6 (14.29)	0.077	0.782
Albumin ≤30 g/L (%)	17 (56.67)	16 (38.10)	2.431	0.119
Mother's age <20 or>35	3 (10.00)	8 (19.05)	0.518	0.472
Gestational hypertention	8 (26.67)	11 (26.19)	0.002	0.964
Gestational diabetes	5 (16.67)	8 (19.05)	0.067	0.796
Corticosteroid use before birth	12 (40.00)	11 (26.19)	1.535	0.215
Previous abortions ≥2	5 (16.67)	8 (19.05)	0.067	0.796
Oligohydramniosis	3 (10.00)	1 (2.38)	——	0.301^F^
Placenta abruption	2 (6.67)	3 (7.14)	——	1.000^F^
Placenta previa	4 (13.33)	1 (2.38)	——	0.153^F^
NRDS	14 (46.67)	11 (26.19)	3.237	0.072
Neonatal pneumonia	30 (100.00)	37 (88.10)	——	0.071^F^
VAP	9 (30.00)	3 (7.14)	6.583	0.010
Pneumothorax	2 (6.67)	1 (2.38)	——	0.567^F^
CNS infection	4 (13.33)	1 (2.38)	——	0.153^F^
PVH-IVH (I–II)	9 (30.00)	12 (28.57)	0.017	0.895
PVH-IVH (III–IV)	6 (20.00)	0 (0.00)	——	0.004^F^
PVL	7 (23.33)	3 (7.14)	——	0.082^F^
hs-PDA	8 (26.67)	1 (2.38)	——	0.003^F^
PNAC	7 (23.33)	1 (2.38)	——	0.008^F^
Liver function damage	6 (20.00)	2 (4.76)	——	0.060^F^
Neutropenia	3 (10.00)	7 (16.67)	0.650	0.420
Anemia	28 (93.33)	32 (76.19)	3.703	0.054
ROP	13 (43.33)	4 (9.52)	11.091	0.001
Hypoglycemia	9 (30.00)	9 (21.43)	0.686	0.408
Electrolyte disturbance	24 (80.00)	16 (38.10)	12.446	0.000
Invasive ventilation (%)	14 (46.67)	5 (11.90)	10.886	0.001
Duration of invasive ventilation (d)	6.50 (2.75,9.00)	6.00 (4.00,6.50)	405.500	0.607
Normal frequency on ventilator (%)	13/14 (92.86)	5/5 (100.00)	——	1.000^F^
High frequency on ventilator (%)	6/14 (42.86)	1/5 (20.00)	——	0.603^F^
Days of FiO_2_ >0.4 (d)	2.00 (0.00,7.25)	0.00 (0.00,1.25)	407.500	0.004
Blood transfusion ≥3 times (%)	23 (76.67)	18 (42.86)	8.159	0.004
Time when enteral nutrition starts (d)	5.00 (3.00,7.25)	2.00 (1.00,2.00)	241.500	0.000
The age that infants achieved 120 kcal/kg.d via enteral feeding ≥40 days after birth (%)	23 (76.67)	11 (26.19)	19.397	0.000
Rate of weight gain (g/kg.d)	1.42 (1.07,1.71)	1.46 (1.28,1.84)	518.000	0.201

* *Preterm infants <32 weeks with minimum hospital stays of 28 days*.

*Rate of weight gain (g/kg •d) = (weight at discharge – weight at admission)/(days at hospital × weight at birth)*.

F *Fisher's exact test*.

—— *Not calculated*.

Electrolyte disturbances (OR = 11.024; 95% CI: 2.472–49.167), hs-PDA(OR = 28.530; 95% CI: 1.915–425.039) and the age that infants achieved 120 kcal/kg.d via enteral feeding ≥40 days after birth (OR = 17.652; 95% CI: 2.472–49.167) were identified as the risk factors of BPD devement by the stepwise multiple logistic regression (Table 2).

**Table 2:** Risk factors for BPD based on stepwise multiple logistic regression analysis.

**Variable**	**β**	**S.E**	**χ^2^**	***P***	**OR**	**95%CI**
Electrolyte disturbances	2.400	0.763	9.899	0.002	11.024	2.472–49.167
hs-PDA	3.351	1.378	5.912	0.015	28.530	1.915–425.039
The age that infants achieved 120 kcal/kg.d via enteral feeding ≥40 days after birth	2.871	0.749	14.707	0.000	17.652	2.472–49.167

*Electrolyte disturbance includes hyponatremia, hypernatremia, hypokalemia, hyperkalemia, hypocalcemia, hypercalcemia, hypochloridemia, and hyperchloremia. hs-PDA refers to confirming the opening of the ductus arteriosus (DA) by echocardiography. And there is a significant change in hemodynamics*.

### Fluid and Caloric Intake in BPD and Non-BPD Preterm Infants

The oral intake of fluid and caloric of preterm infants with BPD was significantly lower than those of without BPD on day 3, 7, 14, and 28 after birth (*p* < 0.05). On day 14, their intravenous fluid intake, and, on day 28, both intravenous fluid and caloric intake were higher than those of the non-BPD group (*p* < 0.05). The difference of weight gain between the BPD group and the non-BPD group was insignificant (*p* > 0.05; Table 3).

**Table 3:** Enteral and parenteral nutrition and fluid intake.

**Days after birth**	**BPD group (*n* = 30)**	**Non-BPD group (*n* = 42)**	**t/U**	***P***
**Oral fluid (ml/kg.d)**				
3d	0.00 (0.00,0.00)^a^	4.64 (0.00,13.59)	347.000	0.000
7d	1.33 (0.00,6.62)	10.72 (4.25,26.90)	336.000	0.001
14d	9.47 (1.97,30.94)	27.18 (8.71,70.44)	416.000	0.014
28d	44.46 (25.28,96.41)	104.13 (64.48,143.93)	357.000	0.002
**Intravenous fluid (ml/kg.d)**				
3d	120.31 ± 17.11	115.14 ± 21.24	−1.101	0.275
7d	144.24 ± 21.35	139.53 ± 21.79	−0.911	0.356
14d	144.16 ± 35.64	118.49 ± 44.36	−2.622	0.011
28d	101.75 ± 42.25	48.03 (11.12,108.10)	327.000	0.001
**Total fluid (ml/kg.d)**				
3d	122.16 ± 17.25	122.20 ± 20.99	0.008	0.994
7d	152.91 ± 21.12	160.66 ± 14.83	1.832	0.071
14d	165.37 ± 22.66	160.90 ± 14.58	−1.019	0.312
28d	159.67 ± 24.64	159.30 ± 22.08	−0.066	0.948
**Oral caloric (kcal/kg.d)**				
3d	0.00 (0.00,0.00)^b^	3.17 (0.00,10.35)	374.000	0.001
7d	1.33 (0.00,6.62)	10.72 (4.25,26.90)	336.000	0.001
14d	6.15 (1.28,21.55)	20.52 (7.05,57.05)	406.000	0.010
28d	32.12 (17.19,78.09)	83.76 (51.07,110.94)	367.500	0.003
**Intravenous caloric (kcal/kg.d)**				
3d	54.03 ± 12.53	56.09 ± 14.29	0.633	0.529
7d	71.36 ± 15.99	74.20 ± 14.81	0.776	0.441
14d	71.40 ± 17.61	65.75 ± 23.66	−1.107	0.272
28d	56.82 ± 22.86	28.20 (1.99,56.45)	348.000	0.001
**Total caloric (kcal/kg.d)**				
3d	55.41 ± 14.23	61.43 ± 16.54	1.613	0.111
7d	77.84 ± 18.60	91.17 ± 17.22	3.135	0.003
14d	87.66 ± 18.49	99.71 ± 17.81	2.785	0.007
28d	102.52 ± 22.47	111.73 ± 18.66	1.895	0.062
**Rate of weight gain**				
3d	−4.38 (−6.22,−1.62)	−3.50 (−5.84,−1.02)	585.000	0.607
7d	−4.33 (−8.17,1.31)	−1.45 (−5.11,1.55)	518.000	0.201
14d	5.02 (0.00,10.05)	9.52 (3.18,15.28)	470.000	0.068
28d	32.47 ± 14.55	35.74 ± 12.40	1.025	0.309

a *25 and 75% quartile ranges are presented in the brackets; the maximal value is 23.53*.

b *25 and 75% quartile ranges are presented in the brackets; the maximal value is 15.29*.

*Rate of weight gain (%)= weight (d_X_−d_1_)/d_1_ × 100%, x = 3,7,14,28*.

### Serum sB7-H3 Levels in Preterm Infants

Serum sB7-H3 levels peaked on day 1 in the BPD group and decreased to nadir on day 14, while serum sB7-H3 levels peaked on day 1 and lowest on day 7 in the non-BPD group. On days 1, 7, and 14, serum sB7-H3 levels were all higher in the BPD group compared to the non-BPD group, but only levels on day 7 had statistical significance (*p* < 0.05; Table 4).

**Table 4:** Time-dependent serum expression of sB7-H3.

**sB7-H3 (ng/ml)**	**BPD group (*n* = 30)**	**Non-BPD group (*n* = 42)**	***t***	***P^a^***
1d	47.21 ± 20.11	39.67 ± 18.87	−1.627	0.108
7d	35.59 ± 10.38^*^	26.15 ± 7.73	−4.422	0.000
14d	30.68 ± 10.31^*^	28.14 ± 8.02	−1.177	0.243
28d	31.13 ± 9.44^*^	31.64 ± 10.36	0.213	0.832

* *BPD group, compared to day 1, P < 0.05*.

a *The p-values for BPD vs. non-BPD*.

The serum levels of sB7-H3 on days 1, 7, 14, and 28 did not have a significant difference among the different BPD groups classified by severity (Appendix 2).

### Serum IL-18 Levels in Preterm Infants

The IL-18 serum levels were lowest on day 1 in both the BPD and non-BPD groups. The levels first increased and then gradually decreased in both groups. In the BPD group, the peak IL-18 level was observed on day 14, while the peak IL-18 level was observed on day 7 in the non-BPD group. On days 1, 7, and 14, the IL-18 levels were higher in the BPD group compared to the non-BPD group, but only on day 14 the difference reached statistical significance (*p* < 0.05; Table 5).

**Table 5:** Time-dependent serum expression of IL-18.

**IL-18 (pg/ml)**	**BPD group (*n* = 30)**	**Non-BPD group (*n* = 42)**	***t*/U**	***P***
1d	130.55 ± 119.45	99.63 (41.59,217.28)	615.000	0.864
7d	213.60 ± 158.17^*^	168.84 ± 103.95	−1.355	0.182
14d	292.63 ± 207.13^*^	133.99 (94.69,214.20)	386.000	0.005
28d	158.46 (84.40,302.36)^*^	99.45 (67.33,182.91)	467.000	0.063

* *BPD group, compared to day 1, P < 0.05*.

The serum levels of IL-18 on days 1, 7, 14, and 28 did not have a significant difference among the different BPD severity groups (Appendix 3).

### NCIS of BPD and Non-BPD Preterm Infans

The NCIS score was significantly lower in the BPD group (100.62 ± 7.81) compared to the non-BPD group (88.20 ± 12.99). The NCIS score of preterm infants with mild, moderate, and severe BPD was 101.33 ± 11.55, 88.36 ± 11.34, and 79.60 ± 16.09, respectively. No significant difference was found among the three different severity groups (*F* = 2.994; *p* > 0.05).

### Sensitivity and Specificity of Individual Risk Factors for BPD

**Figure 1:**
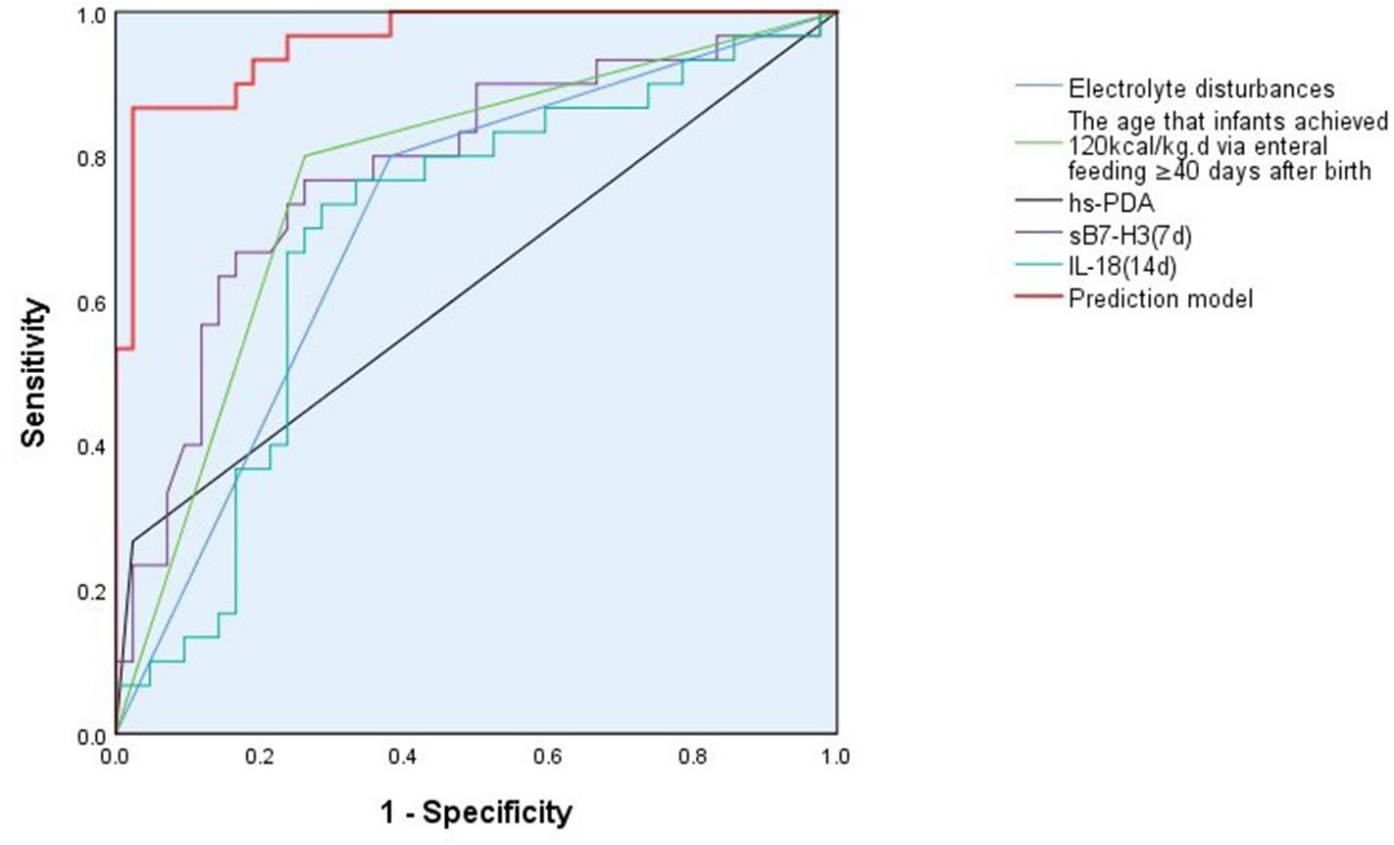
ROC curve for the prediction model for BPD. The prediction model for BPD includes the variables: sB7-H3 (day 7), IL-18 (day 14), NCIS, electrolyte disturbance, hs-PDA, and the age that infants achieved 120 kcal/kg.d via enteral feeding ≥40 days after birth. The AUC, sensitivity and specificity for the prediction model is higher than any of the 6 included risk factors alone.

A ROC analysis, using sB7-H3 (7d), IL-18 (14d), NCIS, electrolyte disturbance, hs-PDA, and the age that infants achieved 120 kcal/kg.d via enteral feeding ≥40 days after birth (and the non-BPD group as a reference), revealed that all these factors can be potential predictors of BPD (Figure 1). The area under the curve (AUC), cut-off value, sensitivity, specificity, and Youden index for these variables are shown in Table 6.

**Table 6:** Sensitivity, specificity, and Youden Index of the independent risk factors.

**Variable**	**Sensitivity (%)**	**Specificity (%)**	**Cut-off**	**AUC**	**95%CI**	***P***	**Standard error**	**Youden index**
sB7-H3 (7d)	76.70	73.80	30.12	0.781	0.670–0.893	0.000	0.057	0.505
IL-18 (14d)	73.30	71.40	167.99	0.694	0.567–0.820	0.005	0.065	0.447
NCIS	73.30	81.00	94.00	0.789	0.676–0.902	0.000	0.058	0.543
Electrolyte disturbances	——	——	——	0.710	0.588–0.831	0.003	0.062	——
hs-PDA	——	——	——	0.621	0.485–0.757	0.081	0.069	——
The age that infants achieved 120 kcal/kg.d via enteral feeding ≥40 days after birth	——	——	——	0.769	0.655–0.883	0.000	0.058	——
Prediction model	86.70	97.60	0.65	0.960	0.919–1.000	0.000	0.021	0.843

——*Not calculated*.

### Sensitivity and Specificity of BPD Prediction Model

sB7-H3 (day 7), IL-18 (day 14), NCIS, electrolyte disturbance, hs-PDA, and the age that infants achieved 120 kcal/kg.d via enteral feeding ≥40 days after birth were brought into the stepwise multiple logistic regression analysis to yeild a predictive model for BPD development. The regression equation was *P* =(3.109 + 0.179X_1_ – 0.001X_2_ – 0.122X_3_ + 1.232X_4_ + 3.805X_5_ + 3.177X_6_). X_1_, X_2_, X_3_, X_4_, X_5_, and X_6_ represent sB7-H3 (7d), IL-18 (14d), NCIS, electrolyte disturbance, hs-PDA, and the age that infants achieved 120 kcal/kg.d via enteral feeding ≥40 days after birth, respectively. P is the predicted probability of logistic regression. The χ^2^-value of the logistic regression prediction model was 60.330 (*p* < 0.001), suggesting that the above 6 variables could significantly explain the development of BPD. Hosmer-Lemeshow was calculated by the classification interaction table (df = 8; *p* > 0.05) indicating that the BPD prediction model fits the observed data well. Using the predictor variables as the test variables, the results showed that the combination of the above six variables with the logistic regression model gives an AUC value of 0.960 in the ROC curve. The sensitivity was 86.70%, and the specificity was 97.60%. The prediction model gave a higher AUC, sensitivity, and specificity than any of the six individual variables alone (Figure 1, Table 7).

**Table 7:** Multifactorial prediction model of BPD.

**Factors**	**β**	**S.E**	**Wals**	***P***	**OR**	**95%CI**
sB7-H3 (7d)	0.179	0.068	6.895	0.009	1.196	1.046–1.366
IL-18 (14d)	−0.001	0.003	0.048	0.827	0.999	0.994–1.005
NCIS	−0.122	0.049	6.226	0.013	0.886	0.805–0.974
Electrolyte disturbances	1.232	0.984	1.566	0.211	3.427	0.498–23.592
hs-PDA	3.805	1.790	4.517	0.034	44.928	1.345–1501.042
The age that infants achieved 120 kcal/kg.d via enteral feeding ≥40 days after birth	3.177	1.021	9.687	0.002	23.974	3.242–177.265

## Discussion

BPD is a multifactorial induced disease affected by antenatal, postnatal as well as genetical factors. Known risk factors include maternal smoking, preeclampsia, intrauterine growth restriction, chorioamnionitis, perinatal hypoxia, mechanical ventilation, oxygen supplementation after birth, perinatal and postnatal infections, and the presence of cardiovascular comorbidities (such as ventricular dysfunction, intracardiac shunts, pulmonary vein stenosis, etc.) (23, 24). And at 36 weeks PMA, the incidences of major neonatal morbidities are higher in infants with BPD than in infants without BPD (25).

The present study collected extensive clinical data from 30 preterm infants with BPD and 42 matching controls. The data collection included the general charactiristics of the infants, maternal conditions, birth injuries, conditions and comorbidities present in the infant, treatment modalities applied during hospitalization of the preterm infant, feeding of the preterm infant, and NCIS on the admission day of the preterm infant. The results revealed that the presence of hs-PDA, electrolyte disturbances, and the age that infants achieved 120 kcal/kg.d via enteral feeding ≥40 days after birth was independent risk factors for the development of BPD.

The finding that PDA is a risk factor for BPD is consistent with existing literature (26). PDA is present in up to 70% of preterm infants, but the causal effect of PDA on BPD is yet to be demonstrated. The persistent left-to-right shunt in hs-PDA leads to pulmonary edema and even hemorrhage (27). The need for mechanical ventilation and oxygen supplementation after birth is therefore higher in infants with hs-PDA. One hypothesis is that mechanical ventilation and oxygen supplementation leads to pulmonary damage and dysfunction in lung microvasculature and thereby inhibits alveolar development, leading to the characteristic impaired alveolar structure seen in BPD (28).

The present study found that the use of invasive ventilation, the number of days with FiO_2_ >0.4, and the percentage of patients diagnosed with VAP were significantly higher in the BPD group compared to the non-BPD group. This is consistent with what is reported in the literature (29). Mechanical ventilation causes volutrauma to the lungs by excessive stretching of the lung tissue and atelectrauma by repeated re-opening of closed regions of the lung. Moreover, injury to the blood-gas barrier causes leakage of fluid and proteins into the alveolar spaces, interfering with surfactant function (10, 30). A high level of oxygen supplementation itself can also be toxic. According to a randomized controlled trial, preterm infants with 30 or 90% oxygen during resuscitation demonstrate a lower risk for BPD in the 30% oxygen group (31).

In the logistic regression analysis, the incidence of hs-PDA was one of the risk factors for BPD. In animal models of BPD using preterm baboons, pharmacologic closure of PDA using ibuprofen is associated with improved alveolar development (32). While early surgical ligation of PDA has recently been shown to be an independent risk factor for the development of BPD (26, 33). Studies in baboons support the perspective that surgical ligation may in fact produce detrimental effects on lung development in preterm infants (34). Infants treated with prostaglandin inhibitors for PDA have lower rates of BPD and mortality than those treated with surgical ligation (35). It is speculated that the different effects of pharmacological and surgical closure of PDA on the development of BPD may be related to the anti-inflammatory effects of the medications (ibuprofen or indomethacin) used to close the ductus (32). Identifying PDA as an independent risk factor for BPD supports the clinical praxis of screening all preterm infants for PDA using bedside cardiac echocardiography. Thus, early identification and treatment for PDA may decrease pulmonary injury and the need for mechanical ventilation and also prevent the development of BPD.

An electrolyte disturbance is also identified as an independent risk factor for the development of BPD. Electrolytes are of crucial importance in maintaining intracellular and extracellular fluid balance and the normal function of cells. As the present study is observational, it is impossible to say if electrolyte imbalance actually causes BPD. Nevertheless, the present results indicate the importance of close monitoring and correction of serum electrolyte levels in preterm infants.

Nutrition is critical for lung development. A previous study suggests that high daily fluid intake and less weight loss during the first 10 days of life increase the risk of BPD in extremely low birth weight infants (36). The pathogenesis behind this is unclear. It is suspected that a high fluid intake preventing weight loss in preterm infants may lead to pulmonary edema which worsens lung function. However, another study finds that an increase in the percent of weight loss is instead a risk factor for BPD and suggest gentle restriction of fluid administration, and the prevention of undernutrition is important in the prevention of BPD (37). Our study showde that preterm infants in the BPD group start enteral nutrition much later than the non-BPD group. Their oral intake of fluid and caloric were also much less than the non-BPD group. On days 7 and 14, the total caloric intake was significantly lower in the BPD group than the non-BPD group, suggesting that both enteral and parenteral nutrition affect the development of BPD. The median age for reaching goal energy intake (120 kcal/kg.d) by enteral feeding is 40 days in all of the 72 preterm infants included in the study. Therefore, reaching goal energy intake by enteral feeding more than 40 days was investigated as a potential risk factor for BPD. The percentage of the age that infants achieved 120 kcal/kg.d via enteral feeding ≥40 days after birth was significantly higher in the BPD group than the non-BPD group, and logistic regression confirms that this was an independent risk factor for BPD. Zhu et al. (38) found that incorporating ω-3 fish oil in a parenteral nutrition emulsion can prevent endoplasmic reticulum stress-induced by total parenteral nutrition therapy. Therefore, improvement and optimization of the formula of total parental nutrition given may improve the outcome of preterm infants and decrease BPD.

Brain injury in preterm infants mainly includes intracranial hemorrhage and cerebral white matter injury, with PVH/IVH and PVL being the most prevalent (39). Both mild and severe PVH/IVH have been found to be correlated with a moderate-to-severe neurodevelopmental impairment such as cerebral palsy and cognitive delay in a meta-analysis (40). The present study found that PVH/IVH (stage III-IV) is significantly more common in the BPD group compared to the non-BPD group. But logistic regression did not show this as an independent risk factor. This suggests that PVH/IVH is related to BPD, but most likely as a confounding factor.

B7-H3 is a new member of the B7 superfamily and has been shown to play a key role in the regulation of T cell-mediated immune responses and also innate immunity by augmentation of lipopolysaccharides and bacterial lipoprotein-induced NF-kappaB activation and proinflammatory cascade via TLR 2 and 4 (11, 41). sB7-H3 levels have been found to be elevated in cerebrospinal fluid and the plasma of children with bacterial meningitis, which have been suggested to be a useful marker in distinguishing bacterial from aseptic meningitis in children (42). In the present study, sB7-H3 was highest on day 1 after birth and the levels gradually decreased in both the BPD and non-BPD groups. The rate of decrease was slower in the BPD group and on day 7 after birth. Preterm infants in the BPD group displayed significantly higher levels of sB7-H3 in the serum compared to the non-BPD group. sB7-H3 levels did not differ depending on BPD severity. Therefore, active monitoring of serum sB7-H3 levels may aid in the early prediction of BPD development but cannot predict the severity of BPD. The ROC curve suggested that the serum sB7-H3 level on day 7 (cut-off value 30.12 ng/ml) only had moderate sensitivity (76.60%) and specificity (73.80%) in predicting the development of BPD.

IL-18 is a member of the IL-1 cytokine family and an important proinflammatory mediator with special importance in pulmonary infection and inflammation. IL-18 has been proposed to be a novel biomarker of ARDS in humans, and its plasma level has been shown to be correlated to ARDS severity and mortality (13). In murine models, mechanical ventilation is shown to enhance IL-18 levels in the lung, serum, and bronchoalveolar lavage fluid, and IL-18-neutralizing antibody treatment reduced lung injury in response to mechanical ventilation (13). Activation of the IL-18 pathway alone has been shown to be sufficient in producing pulmonary inflammation and fibrosis and tissue destruction, similar to that seen in patients with COPD (43). Results from the present study showed that serum IL-18 levels first increased after birth in preterm infants, reaching a peak on day 14 and then the levels started to decrease again. On day 14 after birth, preterm infants with BPD displayed a significantly higher level of IL-18 in the serum compared to those in the non-BPD group. The severity of BPD did not seem to affect the levels of IL-18 in the serum. The dynamic of IL-18 was markedly different from that of sB7-H3, as sB7-H3 levels peak on day 1 after birth and then gradually decrease. This suggests that the serum levels of IL-18 and sB7-H3 are regulated via different mechanisms and are not dependent on each other. The ROC curve suggested serum IL-18 level on day 14 (cut-off value 167.99 pg/ml) only had moderate sensitivity (73.30%) and specificity (71.40%) in predicting the development of BPD.

NCIS is a scoring system for critically ill neonates proposed by the Group of Neonatology of the Chinese Pediatric Society, which is a part of the Chinese Medical Association in 2001. The scoring system is predominately applied clinically in mainland China (44). The present study showed that preterm infants in the BPD group had a significantly higher NCIS score on the day of admission compared to the non-BPD group. The NCIS score did not differ depending on the severity of BPD. The ROC curve suggested that the NCIS score (cut-off value of 94.00) had a rather high specificity (81.00%) but not enough sensitivity (73.30%) in predicting the development of BPD.

BPD is known to be a multifactorial disease that is affected by a complex combination of prenatal, antenatal, and postnatal risk factors, as well as genetics (24). Therefore, the present study aimed to establish a multifactorial prediction model for BPD using a combination of clinical risk factors, comorbidities, nutritional status, serum level of sB3-H7, and IL-18, and NCIS. A prediction model using a combination of the independent risk factors, including serum sB7-H3 (day 7), IL-18 (day 14), electrolyte disturbances, hs-PDA, the age that infants achieved 120 kcal/kg.d via enteral feeding ≥40 days after birth, and NCIS, had an AUC value of 0.960, sensitivity of 86.70% and specificity of 97.60%. The AUC, sensitivity, and specificity of the multifactorial model are markedly higher than using any of the risk factors alone. However, the model can only predict the development of BPD but not the severity of the disease.

The present study is limited by the relatively small cases from a single-center. Although the proposed multifactorial model could predict the development of BPD with relatively high sensitivity and specificity, it could not predict the severity of the disease. Multicenter studies with a large sample size are therefore necessary to produce improved prediction models that can predict and prevent the development of BPD in preterm infants.

## Data Availability Statement

The datasets generated for this study are available on request to the corresponding author.

## Ethics Statement

The studies involving human participants were reviewed and approved by Children's Hospital of Soochow University medical ethics committee. Written informed consent to participate in this study was provided by the participants' legal guardian/next of kin.

## Author Contributions

LD had primary responsibility for protocol development, patient screening, enrollment, outcome assessment, preliminary data analysis, and writing the manuscript. HW participated in the development of the protocol and analytical framework for the study and contributed to the writing of the manuscript. HG, NC, and FH contributed in the same ways as HW and were responsible for patient screening. XuZ and XiZ supervised the design and execution of the study, performed the final data analyses, and contributed to the writing of the manuscript. All authors contributed to the article and approved the submitted version.

## Conflict of Interest

The authors declare that the research was conducted in the absence of any commercial or financial relationships that could be construed as a potential conflict of interest.
