# Supplementary material for: Prediction of Bronchopulmonary Dysplasia in Preterm Infants Using Postnatal Risk Factors
Source: Front Pediatr. 2020 Jun 26;8:349. doi: 10.3389/fped.2020.00349 (PMC7333538; doi:10.3389/fped.2020.00349)
Supplement: Supplementary file 1 [file Data_Sheet_1.docx]

**Appendix**

**Appendix 1.** Supplemental figure: A flow diagram of the patient recruitment


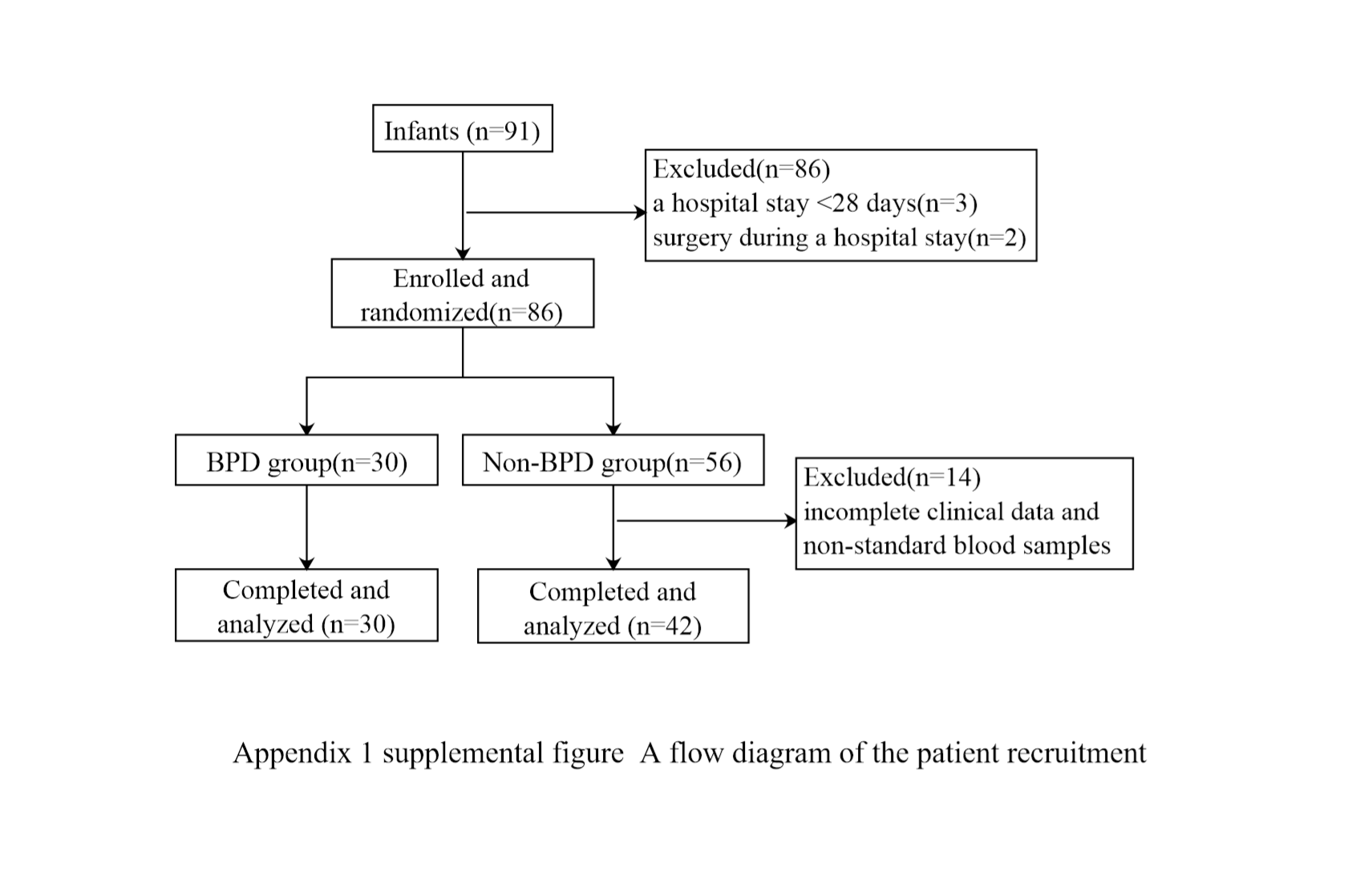


**Appendix 2.** Serum expression of B7-H3 in BPD infants based on disease severity

| sB7-H3 |  | BPD severity |  |  |  |
| --- | --- | --- | --- | --- | --- |
|  | Mild（n=3） | Moderate（n=22） | Severe（n=5） | F | *P* |
| 1d | 40.22±6.44 | 47.45±22.02 | 50.35±17.81 | 0.610 | 0.551 |
| 7d | 32.98±4.65 | 36.36±10.29 | 33.75±14.22 | 0.191 | 0.828 |
| 14d | 33.31±6.40 | 29.60±11.26 | 33.85±7.75 | 0.376 | 0.690 |
| 28d | 31.35±8.19 | 30.82±9.88 | 32.39±9.92 | 0.067 | 0.936 |

**Appendix 3.** Serum expression of IL-18 in BPD infants based on disease severity

| IL-18 (pg/ml) | |  | | BPD severity | |  | |  | |  | |
| --- | --- | --- | --- | --- | --- | --- | --- | --- | --- | --- | --- |
|  | Mild（n=3） | | Moderate（n=22） | | Severe（n=5） | | H/F | | *P* | |  |
| 1 d | 131.39±81.82 | | 98.69（37.67，151.11） | | 143.12±75.18 | | 1.117 | | 0.572 | |  |
| 7 d | 346.53±219.42 | | 193.68±153.65 | | 221.49±133.57 | | 0.546 | | 0.586 | |  |
| 14 d | 371.10±342.54 | | 307.56±208.48 | | 179.88±49.43 | | 0.455 | | 0.639 | |  |
| 28 d | 122.81±50.41 | | 190.82（95.10，340.49） | | 284.27±325.52 | | 0.590 | | 0.745 | |  |
